# Correlation of Different MRI Scoring Systems with Long-Term Cognitive Outcome in Cooled Asphyxiated Newborns

**DOI:** 10.3390/children10081295

**Published:** 2023-07-27

**Authors:** Ok-Hap Kang, Peter Jahn, Joachim G. Eichhorn, Till Dresbach, Andreas Müller, Hemmen Sabir

**Affiliations:** 1 Children’s Hospital, Klinikum Leverkusen, 51375 Leverkusen, Germany; ok-hap.kang@klinikum-lev.de (O.-H.K.); peter.jahn@klinikum-lev.de (P.J.); joachim.eichhorn@klinikum-lev.de (J.G.E.); 2 Department of Neonatology and Pediatric Intensive Care, Children’s Hospital University of Bonn, 53127 Bonn, Germany; till.dresbach@ukbonn.de (T.D.); a.mueller@ukbonn.de (A.M.)

**Keywords:** perinatal asphyxia, magnetic resonance imaging, scoring system, neurodevelopmental outcome, therapeutic hypothermia, prognosis

## Abstract

(1) Background: Cerebral MRI plays a significant role in assessing the extent of brain injury in neonates with neonatal encephalopathy after perinatal asphyxia. Over the last decades, several MRI scoring systems were developed to enhance the predictive accuracy of MRI. The aim of this study was to validate the correlation of four established MRI scoring systems with cognitive long-term outcomes in cooled asphyxiated newborns. (2) Methods: Forty neonates with neonatal encephalopathy treated with therapeutic hypothermia were included in this retrospective study. The MRI scans from the second week of life were scored using four existing MRI scoring systems (Barkovich, NICHD, Rutherford, and Weeke). The patients’ outcome was assessed with the Bayley Scales of Infant Development (BSID-III) at the age of 2 years. To evaluate the correlation between the MRI scoring system with the cognitive scores of BSID-III, the correlation coefficient was calculated for each scoring system. (3) Results: All four MRI scoring systems showed a significant correlation with the cognitive scores of BSID-III. The strongest correlation was found between the Weeke Score (r^2^ = 0.43), followed by the Rutherford score (r^2^ = 0.39), the NICHD score (r^2^ = 0.22), and the Barkovich score (r^2^ = 0.17). (4) Conclusion: Our study confirms previously published results in an independent cohort and indicates that the Weeke and Rutherford scores have the strongest correlation with the cognitive score of BSID-III in cooled asphyxiated newborns.

## 1. Introduction

Hypoxic-ischemic encephalopathy (HIE) due to perinatal asphyxia in newborns is a neonatal disease of worldwide relevance, with an incidence of 1 to 8 per 1000 live births [1,2]. As described in the large randomized controlled trials, a high proportion of affected newborns are left with the remaining sequelae of HIE, including neurodevelopmental impairments such as cerebral palsy, hearing impairment, blindness, or even death despite hypothermia treatment [3,4]. Therapeutic hypothermia is the only established neuroprotective therapy for neonates with moderate-to-severe HIE, as it has been proven to lower the risk of mortality and morbidity. Neonates that show signs of moderate-to-severe HIE are cooled within the first 6 h postnatally at 33–34 °C for 72 h [3,5,6,7,8].

In addition to cranial ultrasound, magnetic resonance imaging (MRI) is widely used to assess the extent of brain injury due to perinatal asphyxia. MRI is the method of choice for the neuroimaging of neonates with HIE. MRI findings are often used as a potential predictor of prognosis for the neurodevelopmental outcomes of infants with neonatal moderate-to-severe HIE [9,10,11]. Therapeutic hypothermia is able to significantly reduce brain injury in term neonates with HIE, and the MRI’s prognostic power is not altered by cooling [12].

The pattern of brain injury is related to the duration and severity of the hypoxic-ischemic event. Acute severe asphyxia results predominantly affect the metabolically active areas, such as the basal ganglia, thalami, brain stem, hippocampus, corticospinal tracts, and sensorimotor cortex, whereas mild-to-moderate chronic asphyxia leads to insults of the white matter and cortex involving the parasagittal watershed areas primarily [13,14]. In addition, studies show that specific patterns of injury are linked with different clinical presentations. The involvement of the basal ganglia, thalami, and posterior limb of the internal capsule (PLIC)—the basal ganglia/thalamus pattern—shows a poor motor outcome, and children are likely to develop cerebral palsy [15,16,17]. Neonates with a watershed predominant pattern of injury are more vulnerable to cognitive and language deficits [18].

Therefore, MRI scoring systems are of high importance when it comes to standardized classification of the extent of brain injury in neonates with moderate and severe HIE. Several scoring systems have been developed to assess the potential impact of perinatal asphyxia on MR imaging. The four most commonly used MRI scoring systems in cooled asphyxiated newborns are the Barkovich score, NICDH score, Rutherford score, and Weeke score [11,12,19,20].

The objective of this study was to evaluate and validate the correlation of the four different MRI scoring systems (Barkovich, NICDH, Rutherford, and Weeke) with 2-year neurodevelopmental cognitive outcome, using the Bayley Scales of Infant and Toddler Development, 3rd edition [21], among cooled neonates with moderate-to-severe HIE in a single local cohort.

## 2. Materials and Methods

### 2.1. Patient Selection

Cerebral MRI images from neonates who were cooled following perinatal asphyxia at the level III (highest level of care) neonatal intensive care unit of the Children´s Hospital of Leverkusen/Germany born between 2010 to 2020 were retrospectively reviewed. All patients underwent whole-body therapeutic hypothermia for 72 h with a rectal temperature of 33.5 ± 0.5 °C, starting within the first 6 h of life. The inclusion criteria for therapeutic hypothermia were as follows:

- Gestational age ≥ 36 + 0 weeks;
- ≤6 h of age;
- pH ≤ 7.0 or base excess ≤ −16 in cord blood or blood sample within the first hour of life;
- Clinical signs of moderate-to-severe encephalopathy after the classification by Sarnat and Sarnat;
- In cases of lacking blood gas analysis, other criteria such as suspected acute perinatal events, e.g., uterine rupture, pathological cardiotocography, and low APGAR score ≤ 5 at 10 min or need for ventilatory support or resuscitation for ≥10 min were included.

The rewarming phase started after 72 h with a gradual rewarming of 0.2–0.4 °C per hour.

The clinical characteristics of eligible infants were obtained using electronic medical records.

All surviving patients underwent at least one cerebral MRI scan in the first two weeks of life before discharge. In this study, only the first MRI of a patient was considered relevant (between days 5 and 19 after birth). Clinical MRI scans were performed on a 1.5 Tesla MRI scanner (Siemens) using established protocols, including T1-, T2-weighted sequences, diffusion-weighted imaging (DWI) sequences, and apparent diffusion coefficient (ADC) mapping.

At the age of 2 years, the neurodevelopmental assessment was performed using the cognitive composite score of the Bayley Scales of Infant and Toddler Development, 3rd edition, by trained pediatricians. A score of >85 was considered to be normal with a mean of 100 (SD of 15), a score of 70 to 85 (−1 SD below mean) was regarded as moderate developmental delay, and a score of <70 (−2 SD below mean) was defined as severe developmental delay. The scores were obtained from the medical charts.

### 2.2. MRI Scoring

The MRI scans were reviewed and scored by two individuals who were blinded to the clinical outcomes. The MRI scoring was performed by an experienced neonatologist (1) with extensive experience in MRI scoring (>10 years of experience) and one pediatrician (2), who underwent intensive training (6 months) by a neuroradiologist and (1) before assessment. The scoring points were individually given and the results were compared. In cases of disagreement, discrepancies were resolved by consensus.

Four different scoring systems were used as follows:

1. Barkovich Score

The most widely used scoring system by Barkovich et al. is the basal ganglia/watershed (BG/W) score, which assesses injury in deep grey nuclei and watershed regions. This score consists of 5 severity categories. (Score 0 = normal; Score 1 = abnormal signal in basal ganglia or thalamus; Score 2 = abnormal signal in the cortex; Score 3 = abnormal signal in the cortex and basal nuclei; Score 4 = abnormal signal in the entire cortex and basal nuclei) It applies to all scoring systems, and a higher score indicates more extensive damage.

The study by Barkovich et al. has shown that it correlates with neuromotor outcomes at 3 and 12 months and cognitive outcomes at 12 months in asphyxiated newborns. A significant association was found between the BG, W, and BG/W scores (first-echo T2 weighted images) and the motor outcome of the Bayley Scales [19]. In addition, it was shown that the Barkovich score was also correlated with long-term outcomes in cooled asphyxiated newborns [22].

2. NICHD score

The scoring system developed by the National Institute of Child Health and Human Development (NICHD) Neonatal Research Network consists of 6 severity grades as follows: Score 0: normal; 1A: minimal cerebral lesions in frontal and parietal subcortical areas; 1B: more extensive cerebral lesions in frontal, parietal, and occipital subcortical areas; 1A and 1B are characterized with no involvement of basal ganglia, thalamus, ALIC, or PLIC; 2A: any BGT, ALIC, or PLIC involvement or watershed infarction without any other additional cerebral lesions; 2B: 2A with additional cerebral lesions; 3: cerebral hemispheric devastation.

This scoring system is known to have a strong correlation with the outcome of death or disability at 18–22 months of age among cooled and non-cooled asphyxiated newborns. Gaining one point in the pattern of brain injury leads to increasing the odds of death or disability by two times [11].

3. Rutherford score

Cerebral lesions in the following areas are analyzed using the scoring systems developed by Rutherford et al.: posterior limb of the internal capsule, basal ganglia and thalamus, white matter, and cortical involvement. Each category is subdivided into normal, mild, moderate, and severe.

PLIC (posterior limb of the internal capsule) score: 0 = normal; 1 = equivocal; 2 = loss.

BGT (basal ganglia and thalamic) score: 0 = normal; 1 = mild; 2 = moderate; 3 = severe.

WM (white matter) score: 0 = normal; 1 = mild; 2 = moderate; 3 = severe.

Cortical involvement score: 0 = normal; 1 = mild; 2 = moderate; 3 = severe.

The higher the involvement of each category, the higher the scores added to the final score. The maximum score amounts to 11 points [12].

4. Weeke Score

In a more detailed scoring system developed by Weeke et al., T1 and T2 weighted images, diffusion-weighted images, and proton magnetic resonance spectroscopy (H-MRS) are additionally evaluated to determine the level of brain damage. The MRI score consists of 4 subscores, including grey matter (basal ganglia, thalamus, PLIC, brainstem, perirolandic cortex, and hippocampus), white matter/cortex (including optic radiation and corpus callosum), and cerebellum. Each category is weighted by its degree (0 = no injury; 1 = focal or <50%; 2 = extensive or >50%) and its location (1 = unilateral; 2 = bilateral). Additional scores are added if there is an intraventricular hemorrhage, subdural hemorrhage, or sinovenous thrombosis (score of 1 each if present). The final score represents a summation of all 4 subscores (maximum grey matter subscore 23, maximum white matter subscore 21, maximum cerebellum subscore 8, and maximum additional subscore 3) with 2 additional scores being added when H-MRS is performed in the basal ganglia and thalamus (score of 1 each if present: reduced N-acetyl aspartate (NAA) peak and increased lactate peak). This adds to a maximum score of 57.

As shown, this score demonstrated a significant association between the grey matter and white matter subscores with death or adverse outcomes at 2 years and at school age [20].

### 2.3. Statistical Analysis

Statistical analysis was performed using GraphPad Prism 6 (GraphPad Software, La Jolla, CA, USA). Pearson’s correlation coefficients were calculated to assess the correlation between the different MRI scores and neurodevelopmental outcomes measured by the cognitive composite score of the Bayley Scales of Infant and Toddler Development. Correlation coefficients were categorized into (1) weak correlation (0.2–0.39); (2) moderate correlation (0.4–0.59); (3) strong correlation (0.6–0.79); and (4) very strong correlation (0.8–1). R squared was calculated to analyze the association between the scoring system and the Bayley Scales of Infant and Toddler Development. As described by Cohen et al., the association was described as very weak (r^2^ < 0.02), weak (r^2^ 0.02–0.13), moderate (r^2^ 0.13–0.26), and substantial (r^2^ ≥ 0.26) [23].

A *p*-value of ≤0.05 was considered to be significant. Cronbach alpha was performed to assess the intra-rater agreement by rescoring a subset of 28 MRI scans. The intra-rater agreement was performed by the reader (2). The scoring date was separated in time by more than two months (time between the scorings). The number of patients who were rescored was randomly selected.

Each scoring system was categorized into 3 severity grades (mild, moderate, and severe) of MRI abnormalities by dividing the maximum possible points of each MRI score into three subgroups. For example, for the Rutherford score, a mild grade was defined by 0 to 3 points, a moderate grade by 4 to 7 points, and a severe grade by 8 to 11 points, as the maximum of 11 points was divided into three subgroups. Abnormalities were detected to study the frequency of detecting abnormalities using the different scoring systems. Every given point results in “detection”, and zero points in each scoring system means “no detection”. An exception was processed for the Weeke score as this is known to be of non-linear distribution, as shown in the study by Szakmar et al. [24].

## 3. Results

### 3.1. Patients Characteristics

A total of 48 newborns underwent therapeutic hypothermia due to moderate-to-severe HIE from 2010 to 2020 in our hospital. Eight patients were excluded from this study: 4 died in the early neonatal period prior to MR imaging, 1 patient was transferred to another hospital without undergoing MRI, 2 patients did not meet the inclusion criteria for this protocol (1 case of apparent life-threatening event on day 14 of life; 1 case of postoperative asphyxiation), and 1 patient´s MRI was not available.

Patient characteristics are presented in Table 1. Thirty-three of the eligible 40 patients were term neonates with a gestational age of >37 + 0 weeks, 7 were preterm neonates (≥36 + 0 weeks), and the median gestational age was 39 + 0 weeks. Of the 40 neonates, 21 (*n* = 21, 52.5%) neonates had moderate HIE, and 13 (*n* = 13, 32.5%) had severe HIE, followed by the mild HIE group (*n* = 6, 15%), as evaluated using the Sarnat and Sarnat classification.

### 3.2. Assessment of Neurodevelopmental Outcome

All 40 patients were assessed between 23 and 25 months of life using the Bayley Scales of Infant and Toddler Development, 3rd edition. A normal development was assumed to be 100 points, a medium impairment 85 points, and a severe impairment 70 points.

At the age of 2 years, twenty-nine (*n* = 29, 72.5%) infants had a normal outcome with a score of >85 points according to the Bayley Scales, nine (*n* = 9, 22.5%) had a moderate adverse outcome, and two (*n* = 2, 5%) had a severe adverse outcome (Table 2).

### 3.3. MRI Findings

We performed MRI scoring on a total of 40 patients. All MRIs were performed between 5 and 19 days after birth with a median and mean age of 8 days.

The mean score and frequency distribution for each MRI score is presented in Table 3. The intra-rater agreement for the Barkovich score was 0.552, for the NICHD score 0.833, for the Rutherford 0.859, and for the Weeke 0.856.

The most represented group for the Barkovich score (75%, *n* = 30) and NICDH score (47.50%, *n* = 19) was found to be in the moderate group with a score of 2 to 3 (2 = abnormal signal in the cortex; Score 3 = abnormal signal in the cortex and basal nuclei) and 1B to 2A (1B = more extensive cerebral lesions in frontal, parietal and occipital subcortical areas; 2A = any BGT, ALIC, or PLIC involvement or watershed infarction without any other additional cerebral lesions).

In comparison, the group with a normal/mild grade was predominantly represented in the MRI scoring system based on Rutherford (*n* = 28, 70%) and Weeke (*n* = 17, 42.5%).

The most frequent pattern of injury noted in the MRI scans was 3 (*n* = 23, 73.3 %) for the Barkovich score (abnormal signal in basal nuclei and cortex), 3 (*n* = 8, 20%) for the Rutherford score, 2A (*n* = 17, 42.5%) for the NICHD score (any BGT, ALIC, or PLIC involvement or watershed infarction without any other additional cerebral lesions), and 3 (*n* = 5, 12.5%) for the Weeke Score.

There was no difference in the detection of abnormalities in this study between the 4 MRI scores (Barkovich, Rutherford, NICHD, and Weeke; 80%, 80%, 80%, and 82.5%, respectively).

### 3.4. Correlation of Bayley Scales of Infant and Toddler Development 3rd Edition with MRI Scoring Systems

**Figure 1:**
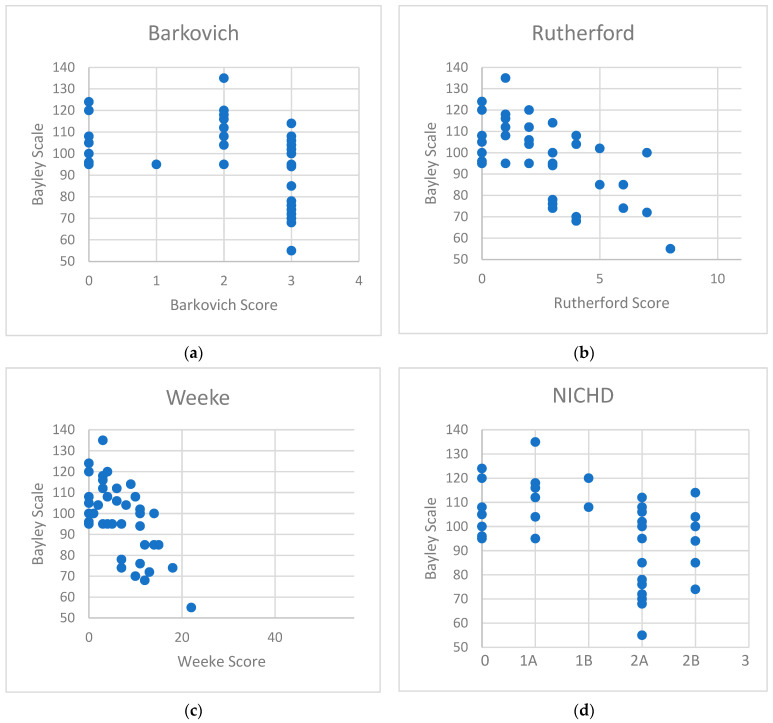
Correlation diagrams of the cognitive scores of Bayley Scales of Infant and Toddler Development with (**a**) Barkovich score, (**b**) Rutherford score, (**c**) Weeke score, and (**d**) NICHD score.

A significant correlation between the cognitive composite score of Bayley Scales of Infant and Toddler Development and all four MRI scoring systems was found. The Weeke (r^2^ = 0.43, *p* < 0.0001) and Rutherford (r^2^ = 0.39, *p* < 0.0001) scores obtained the strongest correlation with the neurodevelopmental outcome at 2 years of age, followed by the NICHD (r^2^ = 0.22, *p* = 0.0018) and Barkovich (r^2^ = 0.17, *p* = 0.0066) scores (Figure 1, Table 4).

## 4. Discussion

The early prediction of neurodevelopmental outcomes in cooled newborns with neonatal encephalopathy still remains a challenge. Nevertheless, it is of significant relevance to provide reliable prognostic information, especially for patients who potentially need early support, e.g., physiotherapy. MR neuroimaging serves as a solid foundation in predicting neurodevelopmental outcomes in cooled asphyxiated newborns [7,10,11,19,25,26,27,28,29]. Therefore, various MRI scoring classifications have been developed over the last decades [11,19,20,30,31,32,33,34]. The reproducibility of research findings in independent cohorts is one of the highest aims in medical research. Therefore, the aim of this study was to investigate the association between four different established MRI scoring classifications (Barkovich, NICHD, Rutherford, and Weeke) and the Bayley cognitive scores in a local cohort of 40 cooled asphyxiated neonates born between 2010 and 2020.

Our findings suggest a significant correlation between all four MRI scoring classifications and the neurodevelopmental cognitive outcome at the age of 2 years. The strongest correlation between Bayley cognitive composite score and MRI score was found for the Weeke score (r^2^ = 0.43, *p* < 0.0001), followed by the Rutherford score (r^2^ = 0.39, *p* < 0.0001), NICHD Score (r^2^ = 0.22, *p* = 0.0018), and the Barkovich score (r^2^ = 0.17, *p* = 0.0066).

All four scoring systems define the deep grey nuclei and white matter/cortex areas as regions of interest. The Rutherford, Barkovich, and NICHD scores use T1 and T2 weighted images, whereas the Weeke score is distinguished by using additional sequences of DWI, ADC, and H-MRS in its assessment. Apparently, the Weeke score is the most comprehensive one and includes other brain abnormalities (e.g., sinovenous thrombosis, intraventricular hemorrhage (IVH), signal abnormalities in cerebellum, brainstem, and corpus callosum), but it remains unclear whether these additional features have additional merit for improving the predictive value of MRI in this patient cohort [20,35]. Martinez-Biarge et al. reported that brainstem lesions were independently associated with death in neonates with HIE and all patients with cerebellar lesions had an extremely poor outcome (63% died, and all 8 survivors had severe cerebral palsy) [27]. In addition, a study by Alderliesten et al. showed that low ADC values of the posterior part of the corpus callosum were associated with an adverse outcome at 18 months in neonates with HIE [9,10,11]. However, Lakatos et al. found that intracranial hemorrhage did not have a significant effect on the outcome of cooled asphyxiated newborns [36].

A more detailed scoring system like the Weeke score allows a broader assessment of the outcome and allows for detecting more abnormalities even in newborns with mild to moderate HIE. Additionally, by using the Weeke score, MRI abnormalities were seen in 50% of neonates with mild HIE in a study by Machie et al., compared to the NICHD (25%) and Barkovich (6%) scores [35].

The strongest correlation between Bayley Scales of Infant and Toddler Development and the different MRI scoring classifications used in our study was found for the Weeke and Rutherford scores. Both scores allow the grading of the extent and severity of each affected area, whereas the Barkovich and NICHD scores bundle the patterns of injury into preformed categories.

In particular, the basal ganglia, PLIC, and thalamus, which are supposed to be the most relevant areas for prediction [15,20,37,38,39], are evaluated by their location (uni-/bilateral) and degree (focal/extensive) in Weeke and Rutherford scores. The descriptions of the extent of the injury, regarding these areas, are not explicitly part of the scoring developed by Barkovich and NICHD.

But these regions seem to provide substantial prognostic information, as in the study by Charon et al., an abnormal PLIC predicted adverse outcomes in neonates with HIE with a sensitivity of 100% (95% CI [54–100]) and specificity of 100% (95% CI [84.4–100]) on MRI performed after 7 days of life [39]. According to Martinez-Biarge et al., severe lesions in the basal ganglia or thalamus have a high predictive value for severe motor impairment (sensitivity of 96%, specificity of 77%, PPV of 85%, and NPV of 94%) [27].

In accordance, the meta-analysis by Ouwehand et al. found that injury in the basal ganglia and thalamus showed a high specificity and PLIC injury implied a high predictive value when MRI was performed in the second week of life [37]. Lally et al. reported that an injury to the basal ganglia/thalamus or PLIC had a sensitivity of 71%, specificity of 88–90%, and area under the ROC curve (AUC) of 0.81–0.82 for predicting adverse outcomes at 2 years [40]. In comparison, lesions in the cortex had a lower sensitivity, yielding a sensitivity of 48%, a specificity of 81%, and an AUC of 0.67 [40].

Although watershed injury appears to have lower sensitivity for adverse outcomes [22,31,37,40], there are several studies that undermine the importance of watershed injuries, as they can be responsible for cognitive dysfunction [15,18,28,29,30,41]. Bach et al. reported a significant association of watershed-predominant injuries with increased odds of an abnormal cognitive outcome (OR 7.01, 95% CI [2.00–24.6]) [28].

The NICHD score does not differentiate between watershed injury and BGT/PLIC injury, as all of them would be classified as 2A/2B (2A: any BGT, ALIC, or PLIC involvement or watershed infarction without any other additional cerebral lesions; 2B: 2A with additional cerebral lesions). In contrast to the other scores, the Barkovich score ranks an injury to the cortex higher than an injury to the basal ganglia/thalamus (Score 1 = abnormal signal in the basal ganglia or thalamus, Score 2 = abnormal signal in the cortex). Furthermore, the assessment of the PLIC, which is known to have high predictive value [16,17,27,42,43], is not specifically included in the Barkovich score [30].

These could be possible reasons that the Weeke and Rutherford scores performed better than the two prior scores in predicting outcomes in our study and could be an explanation for why the predictive value of an item-based scoring system is possibly higher than that of a non-item-based scoring system.

To our knowledge, the study by Bhroin et al. [44] and the recently published study by Langeslag et al. [45] are the only reports to date that compare different MRI scoring classifications with neurodevelopment outcomes assessed by the Bayley Scales of Infant and Toddler Development.

In contrast to our study, Langeslag et al. did not find a statistically significant difference in MRI scoring systems using the Weeke, Rutherford, NICHD, and Trivedi scores or neurodevelopmental outcomes at the age of 2 years. All scoring systems were of high predictive value with comparable AUCs of 0.66–0.71, but surprisingly, the Rutherford score had the lowest predictive value (AUC 0.66) compared to the other three scores. The Weeke score demonstrated the highest AUC (0.71), which is consistent with our findings, but was aware of a restricted comparability, as we did not calculate the AUC in our study and their MRI scans were performed in the first week of life.

Our findings are in agreement with the study by Bhroin et al. They reported a significant association between the Barkovich, NICHD, and Weeke scores and the cognitive composite score of the Bayley Scales of Infant and Toddler Development at 2 years of age. Furthermore, the best correlation was found with the Weeke score, followed by the NICHD score, and lastly, the Barkovich score, which is consistent with our findings.

There is an ongoing debate on whether the predictive value is influenced by the postnatal age when an MRI scan is performed in cooled asphyxiated newborns. In the original publication by Weeke et al. [20], MRI scans were performed early during the first week of life. In contrast, the other MRI scans in the original study by Barkovich et al. [19], NICHD [11], and Rutherford et al. [12] were mostly obtained at the end of the first week and in the second week of life (Rutherford: 2–30 days of life, (mean age 8 days); Barkovich (1–17 days, mean age of 7); NICHD score (mean age of 15 days)).

There are several studies suggesting that MRIs performed in the first week of life (<8 days) have a higher predictive value or higher specificity than MRIs performed later (≥8 days) [20,37,39]. The early MRI heavily relies on using DWI or ADC sequences in its assessment. DWI and ADC abnormalities are seen earlier during the first days, whereas brain injuries on conventional images with T1/T2 weighted images are more subtle in the early days and can be an object of low inter-rater reliability [46] and can lead to the underestimation of the abnormalities. It is often described that T1/T2 weighted changes evolve during the end of the first week and can fully show the extent of injury in the second week of life so MRI scans have more predictive value when performed during the second week of life [11,19,47]. As pseudo-normalization of diffusion-weighted imaging occurs after 6–8 days, the scorer should be aware of it. Although it was reported that in neonates treated with therapeutic hypothermia, this phenomenon occurs later at 10 days of life [32]. Most of our MRI scans were performed in the second week of life (72.5%). Our findings are consistent with prior studies that show an accurate prognostic power for neurodevelopmental outcomes and MRI scans mostly accomplished in the second week of life [11,12,19,22,44,48]. Although the Weeke score was initially developed for an earlier MRI scan, we can confirm that this score is further applicable at a later time point.

On the other hand, numerous publications suggest that the age of the scan does not significantly alter the accuracy of the predictive power of MRI, and scans obtained during therapeutic hypothermia alone allow an accurate view of the extent of the injury as the injuries do not seem to be reversible damages [49,50,51,52,53]. This is relevant for the clinicians in guiding medical decisions who counsel parents and medical staff in the early stages whether to redirect care guardedly or vice versa can presume a favorable outcome when MRI is normal. For most of the centers, the administration of MRI during therapeutic hypothermia can be a challenging task, but various centers have shown that this is feasible without any severe adverse risk [54,55]. The intra-rater agreement for all three MRI scores suggests good reliability, except for the Barkovich score. The reason for this discrepancy is not obvious. Although multiple studies have demonstrated excellent inter-rater agreement using the Barkovich, Rutherford, NICHD, and Weeke scores, there are also studies that underline the potential disagreements between readers. The interpreter has to acknowledge the potential factor of observer error when semi-quantitative scoring is used. In particular, when only conventional MR images from the early days of life are used. Goergen et al. [46] reported a good interobserver agreement (kappa = 0.61–0.69) by using the Griffiths scoring system on DWI images but not for conventional images in a small cohort of nineteen infants. In a recent study by Laptook et al. [56], only a moderate agreement was observed among experienced readers (kappa 0.56) using the NICHD score. As semi-quantitative interpretation can be subject to inter-rater reliability, adding more objective interpretation methods, such as MR spectroscopy or diffusion tensor imaging, can be of prognostic additional value [57,58,59,60,61].

At this point in time, not many NICUs in Germany, including ours, have implemented MR spectroscopy in the MRI standard protocol for newborns with HIE, despite the fact that large evidence of high prognostic accuracy of thalamic proton MRS (AUC 0.99, 95% CI 0.94–1.00) has been described [40]. Further efforts need to be taken to routinely use MR spectroscopy in the standard clinical imaging protocol to provide an additional prognostic value of MR imaging.

There are several limitations to our study. One of the limitations was the small cohort size studied at a single center. Due to the retrospective nature of this study, some of the required data were not available, as a portion of the cohort was lost to follow-up. As the timing of MRI scans differed vastly in our cohort (5 to 19 days of life), the comparability of the scoring could be compromised.

The distinguished relationships between the motor and language composite score and MRI scoring systems could not be reported in our study, as we had a large number of patients in our small cohort that had no data for these Bayley subscales reported. This was due to different reasons and individual decisions by the examiner. Another limitation is the fact that we cannot state any information about the correlation of long-term outcomes with MRI scoring systems in school-aged children, and further studies are needed to compare the MRI scoring systems with a cohort at a later age. Lastly, as the intra-rater agreement for the Barkovich score was suboptimal in our study, under- or overestimation of the results of the Barkovich score cannot be excluded.

## 5. Conclusions

The findings strengthen the prognostic value of MR imaging in asphyxiated newborns treated with therapeutic hypothermia. The strongest correlation between the Bayley Scales of Infant and Toddler Development at the age of 2 years was found between the Weeke and Rutherford scores, followed by the NICHD and Barkovich scores. Future studies need to be conducted to assess the relationships between MRI scoring systems and longer-term outcomes in cooled asphyxiated patients to examine the accuracy of cerebral imaging for longer-term prognostication at school age.

## Figures and Tables

**Table 1:** Patient characteristics.

Variable	Sample
gestational age (days), mean (SD ^1^)	273 (18)
birth weight (g), mean (SD)	3338 (659)
male, *n* (%)	20 (50%)
apgar 1 min, mean (SD)	2 (2)
apgar 5 min, mean (SD)	4 (2)
apgar 10 min, mean (SD)	6 (2)
Sarnat grade, *n* (%)	
mild	6 (15%)
moderate	21 (52.5%)
severe	13 (32.5%)
lowest pH level before the start of HT ^2^, mean (SD)	6.89 (0.16)
lowest BE ^3^ before the start of HT, mean (SD)	−20.19 (18.43)
age at scan, days, mean (SD)	8 (2)

^1^ standard deviation; ^2^ therapeutic hypothermia; ^3^ base excess.

**Table 2:** Bayley cognitive outcome scores.

Bayley score, mean (SD)	97 (17)
normal outcome (score > 86 P)	29 (72.5%)
moderate adverse outcome (score 70 to 85 P)	9 (22.5%)
severe adverse outcome (score < 70 P)	2 (5%)

**Table 3:** MRI score results.

MRI Scoring	
Barkovich, mean (SD)	2 (1)
mild (0 to 1 P)	10 (25%)
moderate (2 to 3 P)	30 (75%)
severe (4 P)	0
Rutherford, mean (SD)	3 (2)
mild (0 to 3 P)	28 (70%)
moderate (4 to 7 P)	11 (27.50%)
severe (8 to 11 P)	1 (2.50%)
NICHD, mean (SD)	2 (1)
mild (0 to 1A)	14 (35%)
moderate (1B to 2A)	19 (47.50%)
severe (2B to 3)	7 (17.50%)
Weeke, mean (SD) normal (0 to 4 P)	7 (6) 17 (42.5%)
mild (5 to 10 P)	11 (27.5%)
moderate (11 to 15 P)	10 (25%)
severe (>15 P)	2 (5%)

**Table 4:** Pearson correlation coefficient (r) and significance (*p*-value).

	Bayley vs. NICHD	Bayley vs. Weeke	Bayley vs. Barkovich	Bayley vs. Rutherford
Pearson r				
r	−0.4785	−0.6604	−0.4224	−0.6278
95% confidence interval	(−0.6875 to −0.1962)	(−0.8061 to −0.4392)	(−0.6485 to −0.1276)	(−0.7856 to −0.3931)
R squared	0.2289	0.4361	0.1784	0.3941
*p* value				
P (two-tailed)	0.0018	<0.0001	0.0066	<0.0001

## Data Availability

All data can be obtained from the corresponding authors.
